# Do Reduction Maneuvers Affect Recurrence in Pediatric Radial Head Subluxation? A Ten-Year Retrospective Analysis

**DOI:** 10.1007/s43465-026-01785-3

**Published:** 2026-04-21

**Authors:** Mehmet Boz, Gülseda Boz, Tarik Altunkılıç, Bünyamin Arı, İsmail Güzel, Ümit Yasin Barbak

**Affiliations:** 1 Department of Orthopedics and Traumatology, Faculty of Medicine, Malatya Turgut Ozal University, Malatya, Turkey; 2 Inonu University Faculty of Medicine Department of Public Health, Malatya, Turkey

**Keywords:** Nursemaid’s elbow, Radial head dislocation, Reduction maneuver, Pediatric trauma

## Abstract

**Background:**

This study aimed to determine whether the choice of reduction maneuver in patients presenting to the emergency department with nursemaid’s elbow influences the likelihood of recurrent dislocation. Additionally, the study sought to describe the epidemiological characteristics of these cases.

**Methods:**

This retrospective study included 1359 patients diagnosed with nursemaid’s elbow who presented to the Emergency Department of Malatya Training and Research Hospital from January 2015 to June 2025. Data on demographics, side affected, radiographic evaluation, reduction maneuver, and recurrence were analyzed. The chi-square test was used for statistical analysis, with p < 0.05 considered significant.

**Results:**

The median age was 2 years, with 61.2% female. Most cases (88.1%) were in the 0–3-year age group. Left-sided involvement accounted for 63.0%, right-sided involvement 37.0%. Hyperpronation was used in 68.4%, supination–flexion in 31.6%. Recurrence rate was 4.8%, higher with right elbows and supination–flexion (p < 0.05).

**Conclusion:**

Nursemaid’s elbow was more common in children under three, particularly girls, and affected the left arm. Recurrence was lower after hyperpronation than after supination–flexion.

**Level of Evidence:**

Retrospective analysis study.

## Introduction

Nursemaid’s elbow (NME), also known as radial head subluxation or "pulled elbow," is a common injury in young children. It usually follows a sudden pull or traction on the extended arm, causing the radial head to slip from under the annular ligament. In children, this ligament is thinner and more elastic than in adults, making it more vulnerable to stretching or displacement. The injury often results in localized pain and reduced limb function [1–3]. Radial head subluxation is a frequent elbow injury in young children, with about 8 cases per 10,000 children aged 6 or younger annually. Most cases occur between 6 months and 6 years, while older children are less affected [4]. Females are affected more often than males, showing a slight but steady predominance. Affected children are usually in higher weight percentiles for their age and sex [5]. The left arm is more often involved, likely due to right-hand dominance among caregivers and children [6]. Pathophysiologically, radial head subluxation typically follows a sudden traction force on an extended, pronated arm, causing the radial head to slip under the annular ligament [1]. However, non-axial mechanisms, such as a fall or direct blow, do not exclude nursemaid’s elbow in young children [7]. Providers usually diagnose the condition based on classic signs, such as the child’s reluctance to use the affected arm and a fixed, slightly flexed, pronated position [8]. A palpable or audible click may indicate a successful reduction [9]. Treatment is usually closed reduction, which involves simple manual manipulation and results in rapid, often immediate symptom relief [10, 11]. This study aimed to determine whether the type of reduction maneuver affects the risk of recurrent dislocation. It also sought to provide a detailed epidemiological profile of nursemaid’s elbow using a retrospective analysis of 10 years of data from children aged 0–6 years who presented to the emergency department trauma unit.

## Methods

### Study Design

This study is a retrospective descriptive analysis.

### Population and Sample

A total of 1371 patients who presented to the Emergency Department of Malatya Training and Research Hospital between January 2015 and June 2025 and were recorded in the hospital electronic database with an ICD-10 diagnosis code of nursemaid’s elbow (radial head subluxation) were initially included in the study. Radiographic examinations of all patients were reviewed, and 12 patients with accompanying fractures were excluded, resulting in a final study population of 1359 patients.

### Data Sources and Inclusion Criteria

#### Inclusion Criteria

- Presentation to the emergency department following trauma or sudden pulling of the elbow,
- Clinically diagnosed with nursemaid’s elbow,
- Age under 6 years.

#### Exclusion Criteria

- Presence of concomitant fracture or other traumatic injury in the elbow,
- Incomplete medical records,
- Previous surgery of the elbow or wrist.

### Data Collection

Patient data, including age, sex, year of presentation, affected side (right/left), reduction method, radiographic status, and recurrence of dislocation, were recorded. For the reduction method, the preferred maneuver for NME was identified from emergency department records of 29 orthopedic surgeons who treated 1359 patients over the 10-year study period; these data were incorporated into the analysis. Data collection.

Statistical analyses were performed using SPSS version 22.0. Medians (min–max) summarize continuous variables; categorical variables are summarized using counts and percentages. Categorical differences were tested using the chi-square. P < 0.05 indicated significance.

### Ethical Considerations

Ethical approval was received from the Clinical Research Ethics Committee of Turgut Özal University (Approval No: 2025/281). The study followed the Declaration of Helsinki.

## Results

A total of 1359 patients with NME were included in the study. The median age was 2 years (range: 0–6 years). The distribution of patients according to sex, age group, and clinical characteristics is presented in Table 1. Of the study population, 61.2% were female (n = 832) and 38.8% were male (n = 527). According to age groups, NME was most frequently observed in children aged 2–3 years (49.9%; n = 678), followed by those aged 0–1 years (38.2%; n = 519), 4–5 years (10.8%; n = 147), and 6 years (1.1%; n = 15). Hyperpronation was used as the reduction maneuver in 68.4% of cases (n = 930), while the supination–flexion maneuver was applied in 31.6% (n = 429). Of 1,345 patients evaluated for recurrence, only 65 (4.8%) experienced recurrent dislocations. Regarding the side of involvement, 63.0% of cases (n = 856) occurred in the left arm, while 37.0% (n = 503) involved the right arm.

**Table 1:** Distribution of patients by sex, age group, and clinical characteristics

Sex	n	%
Male	527	38.8
Female	832	61.2
Age group		
0–1 years	519	38.2
2–3 years	678	49.9
4–5 years	147	10.8
6 years	15	1.1
Radiographic status		
Present	976	72.8
Absent	383	28.2
Reduction Maneuver		
Hyperpronation	930	68.4
Supination–Flexion	429	31.6
Dislocation recurrence		
Yes	65	4.8
No	1294	95.2
Affected side		
Left elbow	856	63.0
Right elbow	503	37.0
n	1359	1359

Table 2 shows clinical characteristics by age group. Among males: 40.4% were 0–1, 46.9% were 1–2, 11.4% were 3–4, and 1.3% were 5–6 years old. For females, the distribution was 36.8%, 51.8%, 10.5%, and 1.0%. No significant difference in age group by sex (p = 0.343). For radiographic evaluation: 37.4% were 0–1, 49.9% were 1–2, 11.4% were 3–4, 1.3% were 5–6 years. Age groups did not impact radiographic use (p = 0.366). Hyperpronation and supination–flexion groups had similar age distributions. The left elbow was most often affected in the 1–2 age group (50.2%), and the right showed a similar distribution (49.3%). Age group and affected side were not significantly associated (p = 0.448).

**Table 2:** Distribution of clinical characteristics according to age groups

	Age group	Age group	Age group	Age group	p
Sex	0-1 years	1-2 years	3-4 years	5-6 years	
Male	213(%40.4)	247(%46.9)	60(%11.4)	7(%1.3)	0.343
Female	306(%36.8)	431(%51.8)	87(%10.5)	8(%1.0)	
Radiographic status					
Present	365(%37.4)	487(%49.9)	111(%11.4)	13(%1.3)	0.366
Absent	154(%40.2)	191(%49.9)	36(%9.4)	2(%0.5)	
Reduction maneuver					
Hyperpronation	366(%39.4)	466(%50.1)	90(%9.7)	8(%0.9)	0.101
Supination–flexion	153(%35.7)	212(%49.4)	57(%13.3)	7(%1.6)	
Dislocation recurrence					
Yes	19(%29.2)	41(%63.1)	4(%6.2)	1(%1.5)	0.136
No	500(%38.6)	637(%49.2)	143(%11.1)	14(%1.1)	
Affected Side					
Left elbow	333(%38.9)	430(%50.2)	85(%9.9)	8(%0.9)	0.448
Right elbow	186(%37.0)	248(%49.3)	62(%12.3)	7(%1.4)	

We assessed recurrence by sex, affected elbow, and reduction maneuver (Table 3). Recurrence was 4.6% (n = 24) for males, 4.9% (n = 39) for females; difference not significant (p = 0.795). Recurrence was higher in right elbows (7.2%, n = 36) than left (3.4%, n = 29) (p = 0.002). Recurrence occurred in 4.0% (n = 32) after hyperpronation, 7.7% (n = 33) after supination–flexion; the latter had significantly higher recurrence (p = 0.001).

**Table 3:** Recurrence according to sex, reduction maneuver, and affected elbow

	Recurrence status p	Recurrence status p	Recurrence status p
Sex	Yes	No	
Male	24(%4.6)	503(%95.4)	0.795
Female	41(%4.9)	791(%95.1)	
Affected side			
Left elbow	29(%3.4)	827(%96.6)	0.002
Right elbow	36(%7.2)	467(%92.8)	
Reduction maneuver			
Hyperpronation	32(%4.0)	898(%96)	0.001
Supination–flexion	33(%7.7)	396(%92.3)	

## Discussion

Our study, as one of the few large-scale epidemiological investigations reported in the literature, provides a comprehensive analysis of nursemaid’s elbow cases presenting to the emergency department, examining patient age, sex, side of involvement, and recurrence. In our study, the vast majority of cases (88.1%) occurred in children aged 0–3 years, whereas only 1.1% occurred in the 5–6-year age group.5,9 These findings indicate that nursemaid’s elbow is rare after the age of three, which is consistent with the literature. Regarding sex distribution, the proportion of females (61.2%) was higher than that of males (38.8%), consistent with previous studies [6, 12]. Some studies suggest that this difference may be attributable to cultural or sociodemographic factors [5, 13]. The predominance of left-sided involvement is a finding frequently reported in the literature. This has been suggested to result from parents typically using their right hand to pull the child’s left arm. In our study, similar results were observed, with the left side being affected more frequently (63%) than the right side (37%). No significant differences were found between the affected side and either age group or recurrence. This finding suggests that side dominance does not appear to be a determining factor in clinical prognosis. 13 In our study, radiographs were obtained in 72.8% of patients; however, no statistically significant association was found between age groups and radiograph utilization. This rate is considerably higher than the approximately 20–50% reported in the literature and may reflect clinicians’ tendency to perform radiographic evaluation when a fracture is suspected. Our findings differ from those of previous studies, emphasizing that radiographs are unnecessary when nursemaid’s elbow can be diagnosed based on typical clinical findings. Therefore, excessive use of radiography in this age group represents a significant concern, potentially leading to unnecessary radiation exposure and increased healthcare costs [14]. All patients underwent a reduction maneuver, with no significant differences observed among age groups regarding its application. The literature reports that the hyperpronation maneuver has higher success rates than the supination–flexion technique [12, 15–17]. In our study, hyperpronation was performed in 68.4% of cases, while the supination–flexion technique was used in 31.6%, a distribution consistent with the literature. On the other hand, no significant association was found between the success of the initial reduction attempt and patient age or sex. This suggests that success in reduction may be more closely related to the chosen technique and the clinician’s experience with it. In our study, the recurrence rate of nursemaid’s elbow was 4.8%, which lies at the lower end of the 5–10% range reported in the literature. In a study by Diab et al. involving 50 cases, recurrence was observed in only three patients. [18] Although no statistical association was found between recurrence and age or sex, the recurrence rate was higher in the right elbow and in cases treated with the supination–flexion maneuver, a statistically significant difference. A possible explanation for the lower recurrence rate observed after hyperpronation may be related to the underlying mechanism of nursemaid’s elbow, which is thought to involve displacement or interposition of the annular ligament after axial traction on the pronated forearm. Hyperpronation may facilitate a more direct repositioning of the displaced ligament and thereby achieve a more complete and stable reduction than the supination–flexion maneuver. This may partly explain the lower recurrence observed in our series. However, because of the retrospective design of our study and the absence of dynamic imaging or biomechanical evaluation, this explanation remains speculative and should be confirmed by prospective studies. It should also be noted that recurrent cases may be more closely associated with individual risk factors, such as ligamentous laxity or familial habits. [2, 19]].

Nursemaid’s elbow is a common cause of emergency department visits in childhood, yet it is often a condition that can be promptly diagnosed and treated with a simple reduction maneuver. However, despite its frequent occurrence in clinical practice, epidemiological data on this condition are limited, particularly in our country. Most studies in the literature have been limited to case reports or small case series; there is a notable lack of large-scale research that systematically evaluates age, sex, side of involvement, reasons for presentation, misdiagnoses, rates of unnecessary imaging, and the relationship between recurrence and the type of reduction maneuver. Our study aims to provide one of the most comprehensive epidemiological datasets on nursemaid’s elbow cases in our country, with a large sample size. The low need for radiological imaging in patients presenting with typical clinical findings suggests that routine radiography is often unnecessary. Furthermore, the low recurrence rates observed after reduction are clinically significant, as they may help prevent excessive parental anxiety and unnecessary investigations. Notably, the success of the reduction appears to be more closely related to the technique used and the clinician’s experience than to patient age or sex. The main strength of this study lies in its detailed analysis of a large patient series, examining parameters such as age, sex, affected side, type of reduction maneuver (hyperpronation vs. supination–flexion), and recurrence. Additionally, the low recurrence rate observed after hyperpronation suggests that this maneuver may help clinicians select the preferred reduction technique. However, the single-center and retrospective design of the study may limit the generalizability of the findings. Future multicenter, prospective studies could provide clearer insights into the effectiveness of different reduction techniques and potential risk factors, thereby offering valuable guidance for clinical practice.

## Limitations

The main limitations of this study include its retrospective design, which relies on the content and detail of clinical notes. Additionally, patients’ congenital connective tissue disorders and ligamentous laxity could not be assessed. It should also be noted that indications for radiography may have varied among physicians.

## Data Availability

The data presented in this study are available from the corresponding author upon request, subject to the following restriction (specify the reason for the limitation).

## References

[1] Paluch, L. K. (2024). Nursemaid’s elbow: Radial head subluxation injuries in children. *Journal of the American Academy of Physician Assistants,**37*(6), 18–21. 10.1097/01.JAA.0000000000000025 38747889 10.1097/01.JAA.0000000000000025

[2] Meadows, M., Vuong, B., Storaci, H., et al. (2024). Biomechanical simulation of radial head subluxation in cadaveric pediatric elbows. *Journal of the Pediatric Orthopaedic Society of North America,**7*, 100036. 10.1016/j.jposna.2024.100036 40433293 10.1016/j.jposna.2024.100036 PMC12088284

[3] Rudloe, T. F., Schutzman, S., Lee, L. K., et al. (2012). No longer a “Nursemaid’s” Elbow. *Pediatric Emergency Care,**28*(8), 771–774. 10.1097/PEC.0b013e3182624906 22858743 10.1097/PEC.0b013e3182624906

[4] Zhou, J. J., Shah, N. V., Scheer, R. C., et al. (2022). Trends and epidemiology of radial head subluxation in the United States from 2004 to 2018. *European Journal of Orthopaedic Surgery & Traumatology.,**32*(6), 1137–1144. 10.1007/s00590-021-03089-8 34363491 10.1007/s00590-021-03089-8

[5] Vitello, S., Dvorkin, R., Sattler, S., et al. (2014). Epidemiology of nursemaid’s elbow. *Western Journal of Emergency Medicine,**15*(4), 554–557. 10.5811/westjem.2014.1.20813 25035767 10.5811/westjem.2014.1.20813 PMC4100867

[6] Wong, K., Troncoso, A. B., Calello, D. P., et al. (2016). Radial head subluxation: Factors associated with its recurrence and radiographic evaluation in a tertiary pediatric emergency department. *Journal of Emergency Medicine,**51*(6), 621–627. 10.1016/j.jemermed.2016.07.081 27687166 10.1016/j.jemermed.2016.07.081

[7] Li, N., Khoo, B., Brown, L., et al. (2021). Nonaxial traction mechanisms of Nursemaid’s Elbow. *Pediatric Emergency Care,**37*(6), e292–e294. 10.1097/PEC.0000000000001963 32149992 10.1097/PEC.0000000000001963

[8] Crowther, M. (2009). Elbow pain in pediatrics. *Current Reviews in Musculoskeletal Medicine,**2*(2), 83–87. 10.1007/s12178-009-9049-4 19468873 10.1007/s12178-009-9049-4 PMC2697336

[9] Nardi NM, Schaefer TJ. *Nursemaid Elbow*.; 2025. 28613528

[10] Bexkens, R., Washburn, F. J., Eygendaal, D., et al. (2017). Response to effectiveness of reduction maneuvers in the treatment of nursemaid’s elbow: A systematic review and meta-analysis. *American Journal of Emergency Medicine,**35*(7), 1016–1017. 10.1016/j.ajem.2017.02.010 28215960 10.1016/j.ajem.2017.02.010

[11] Samargandi, R., Alharthi, R. M., Alghamdi, A. M., et al. (2025). Assessment of population knowledge and awareness of Nursemaid’s Elbow in children: A cross-sectional study in Al-Baha Region, Saudi Arabia. *Journal of Orthopaedic Case Reports,**15*(6), 257–263. 10.13107/jocr.2025.v15.i06.5730 40520758 10.13107/jocr.2025.v15.i06.5730 PMC12159624

[12] Serafı´n Garcıá-Mata S, Hidalgo-Ovejero A. *Efficacy of Reduction Maneuvers For “Pulled Elbow” in Children: A Prospective Study of 115 Cases*.; 2013. www.pedorthopaedics.com 10.1097/BPO.0000000000000130 24322628

[13] Irie, T., Sono, T., Hayama, Y., et al. (2014). Investigation on 2331 cases of pulled elbow over the last 10 years. *Pediatric Reports,**6*(2), 26–28. 10.4081/pr.2014.5090 10.4081/pr.2014.5090 PMC4076648 24987508

[14] Macıas, C., Wıebe, R., & Bothner, J. (2000). History and radiographic findings associated with clinically suspected radial head subluxations. *Pediatric Emergency Care,**16*(1), 22–25. 10.1097/00006565-200002000-00007 10698138 10.1097/00006565-200002000-00007

[15] Ulici, A., Herdea, A., Carp, M., et al. (2019). Nursemaid’s elbow - Supination-flexion technique versus hyperpronation/forced pronation: Randomized clinical study. *Indian Journal of Orthopaedics,**53*(1), 117–121. 10.4103/ortho.IJOrtho_442_17 30905991 10.4103/ortho.IJOrtho_442_17 PMC6394198

[16] Bek, D., Yildiz, C., Köse, Ö., et al. (2009). Pronation versus supination maneuvers for the reduction of “pulled elbow”: A randomized clinical trial. *European Journal of Emergency Medicine,**16*(3), 135–139. 10.1097/MEJ.0b013e32831d796a 19262394 10.1097/MEJ.0b013e32831d796a

[17] Aksel, G., Küka, B., İslam, M. M., et al. (2025). Comparison of supination/flexion maneuver to hyperpronation maneuver in the reduction of radial head subluxations: A randomized clinical trial. *American Journal of Emergency Medicine,**88*, 29–33. 10.1016/j.ajem.2024.11.026 39579408 10.1016/j.ajem.2024.11.026

[18] Diab, H. S., Hamed, M. M. S., & Allam, Y. (2010). Obscure pathology of pulled elbow: Dynamic high-resolution ultrasound-assisted classification. *Journal of Children’s Orthopaedics,**4*(6), 539–543. 10.1007/s11832-010-0298-y 22132031 10.1007/s11832-010-0298-y PMC2981709

[19] Hagroo GA, Zaki HM, Choudhary MT, et.al. *Pulled Elbow-Not the Effect of Hypermobility of Joints*. Vol 7.; 1995. 10.1016/0020-1383(95)00133-6 8745806

